# Synergistic Evaluation of Passive Microwave and Optical/IR Data for Modelling Vegetation Transmissivity towards Improved Soil Moisture Retrieval

**DOI:** 10.3390/s22041354

**Published:** 2022-02-10

**Authors:** Mina Moradizadeh, Prashant K. Srivastava, George P. Petropoulos

**Affiliations:** 1 Department of Geomatics, Faculty of Civil and Transportation Engineering, University of Isfahan, Isfahan 8174673441, Iran; m.moradizadeh@eng.ui.ac.ir; 2 Remote Sensing Laboratory, Institute of Environment and Sustainable Development, Banaras Hindu University, Varanasi 221005, Uttar Pradesh, India; prashant.iesd@bhu.ac.in; 3 Department of Geography, Harokopio University of Athens, 17671 Athens, Greece

**Keywords:** vegetation transmissivity, land surface parameters, microwave remote sensing, *AMSR2*, soil moisture

## Abstract

Vegetation cover and soil surface roughness are vital parameters in the soil moisture retrieval algorithms. Due to the high sensitivity of passive microwave and optical observations to Vegetation Water Content (*VWC*), this study assesses the integration of these two types of data to approximate the effect of vegetation on passive microwave Brightness Temperature (*BT*) to obtain the vegetation transmissivity parameter. For this purpose, a newly introduced index named Passive microwave and Optical Vegetation Index (*POVI*) was developed to improve the representativeness of *VWC* and converted into vegetation transmissivity through linear and nonlinear modelling approaches. The modified vegetation transmissivity is then applied in the Simultaneous Land Parameters Retrieval Model (*SLPRM*), which is an error minimization method for better retrieval of *BT*. Afterwards, the Volumetric Soil Moisture (*VSM*), Land Surface Temperature (*LST*) as well as canopy temperature (*T_C_*) were retrieved through this method in a central region of Iran (300 × 130 km^2^) from November 2015 to August 2016. The algorithm validation returned promising results, with a 20% improvement in soil moisture retrieval.

## 1. Introduction

Volumetric Soil Moisture (*VSM*) content is the volume of water accumulated in soil pores, usually recorded as a percent or volumetric ratio (i.e., cm^3^/cm^3^) for different depths. The knowledge of soil moisture content is essential in several applications in the field of ecological, hydrological and meteorological processes [1]. Predictions and results of these applications are highly dependent on the accuracy of the *VSM* data [2]. Due to high temporal and spatial variability of soil moisture and temperature, remote sensing is the only rational instrument to measure and monitor them in an efficient manner, in a wide range of areas [3,4].

Microwave (*MW*) remote sensing has great potential for measuring and monitoring of soil characteristics. In light of this, different methodologies are found in the literature to explain the rationale behind the estimation of surface soil characteristics accurately [5]. Findings of those studies among others have reported that the radar is more sensitive to surface features, such as roughness and vegetation structure, and radiometer is more sensitive to the near surface *VSM* and Land Surface Temperature (*LST*) [6].

Factors influencing the Brightness temperature (*BT*) of the surface as observed from space, especially in the soil-vegetation medium consist of soil moisture, temperature, vegetation cover (type and amount), surface roughness, soil texture, soil Bulk density (*Bd*) along with elevation, soil depth, soil mineralogy, etc. [7]. A better estimate of those factors is very much needed when assessing their effect on *BT* and soil moisture retrieval [7,8,9]. However, in almost all studies, the quantization or prediction of soil characteristics with a consistent degree of confidence is a challenging task for soil scientists and there exist different soil moisture retrieval algorithms in this context. As the modelling of the soil surface roughness and above-ground vegetation cover effects are critical, the difference in the developed algorithms is in their modelling method with respect to these two parameters [10,11,12]. Other challenges that we are facing in modelling soil characteristics are due to limited penetration of sensors and low sensitivity to the soil parameters [13]. Spatial and temporal resolutions of sensors are another major problem that limits the model’s performance due to mismatch between the ground and satellite footprints [14].

Due to the high temporal dynamics of vegetation effect, its accurate determination is very important. In this study, the relationship between Vegetation Water Content (*VWC*) and vegetation transmissivity, in order to model the effect of vegetation on *BT*, is investigated. The studies by [15] indicate that observations of passive microwave and optical sensors are sensitive to vegetation in different sensor wavelengths. Therefore, in this study, these two types of data are used and integrated under a newly introduced Passive microwave and Optical Vegetation Index (*POVI*) to estimate *VWC* in a more accurate way. Then, with two separate linear and nonlinear modelling approaches, the *POVI* is converted to transmissivity and applied in the Simultaneous Land Parameters Retrieval Model (*SLPRM*) algorithm to estimate the *VSM*, *LST* and canopy temperature (*T_C_*) parameters [16]. Finally, a comparison has been made between the retrieval accuracies of soil surface parameters under linear and nonlinear modelling. The advantage of this algorithm is that it considers the roughness parameter and *VWC*, therefore proposing a solution towards simultaneous retrieval of soil surface parameters. Unlike *VSM* operational retrieval algorithms, *LST* and *T_C_* parameters are not considered equal in order to simplify the algorithm [6].

In purview of the above, this study has been focused on (1) to develop, a new index named as *POVI* for better representation of *VWC*, (2) to assess new index for vegetation transmissivity estimation through linear and nonlinear modelling approaches, (3) to evaluate the modified vegetation transmissivity for retrieval of *BT* through Simultaneous Land Parameters Retrieval Model, (4) to retrieve the volumetric soil moisture, land surface temperature as well as canopy temperature using the improved *BT*.

## 2. Study Area and Datasets

### 2.1. Study Area

**Figure 1:**
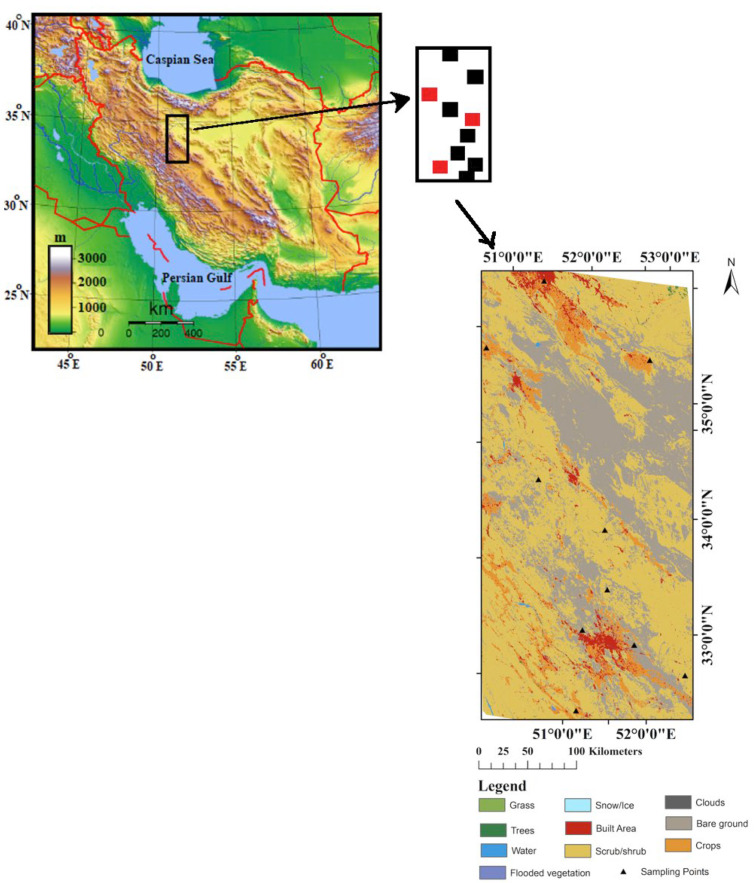
Spatial distribution of the ground stations (Solid black and red squares are locations of calibration and check stations, respectively) and geographic location (Rectangular polygon) of the study region along with the land cover.

In 2016, the data collection experiment was performed in the central Iranian plateau, a semiarid region of Iran located between Isfahan and Tehran provinces (32°43′–35°35′ N, 50°50′–52°10 E) [17]. This region and its ground sites occupy an area of approximately 300 × 130 km^2^, as shown in Figure 1.

This area is generally semi-arid and its average elevation is 1400 m above sea level. As elevation increases from east to west in this region, rainfall and temperature increase and decrease, respectively, which creates different climatic conditions. Based on the meteorological stations’ data, topography is the most important determinant of climate in the study area. Land cover distribution types are diverse in this region, including Grass (steppe species dominate the area). Other cover types that exist in the region are namely Trees, Water bodies, Flooded vegetation, Snow/Ice, Built Area, and Scrub/Shrub. Regarding the potential of this proposed algorithm, 10 sites with different soil moisture content are selected for validation purposes.

### 2.2. Satellite Datasets

In this study, the Advanced Microwave Scanning Radiometer-2 (*AMSR2*) microwave and *MODIS* data registered on 16 dates from November 2015 as well as January, March, May, June and August 2016 from the selected region are applied. The reason for choosing these dates is to make observations in different climatic conditions, and as a result, diversity in the values of soil surface parameters. Details about the dataset used are provided in Table 1. *AMSR2* was launched on the JAXA’s *GCOM-W1* spacecraft in May 2012, replacing the *AMSR*-*E* sensor onboard the NASA’s Aqua satellite. Although the spatial resolution of this sensor has been slightly upgraded, it is still not considered a suitable resolution. This instrument is dual-polarized and measures BT on several frequency channels twice a day. The overpass time of both *MODIS*/*Aqua* and *AMSR2* is about 1:30 A.M. and 1:30 P.M. Because ground data are observed around noon, only daytime *AMSR2* and *MODIS* (or Moderate Resolution Imaging Spectroradiometer) data are used. In addition to having the same overpass time of both the sensors, the other reasons for selecting *MODIS* among other *VIS*/*IR* sensors are its temporal resolution, data availability and good 1 km spatial resolution. The spatial resolution of *AMSR2* in the metric unit is 25 km. The spectral bands 2 and 5 of *MODIS* data are upscaled to 25 km by using the averaging method.

### 2.3. Ground Datasets

*LST* is determined and ground samples are taken concurrent to satellite overpass. *VSM* and *LST* measurements are taken at the depths of 0–6 cm and 0–5 cm, respectively. In the laboratory, *VSM* content is also estimated for the above-mentioned dates. The average *Bd* of the study region is measured by applying a few ground samples in the laboratory. Towards this, according to the means of the region, the values of 1.2 and 0.12 are considered for Bd and surface roughness, respectively. In total, the 10 best stations used in this study have uninterrupted quality-controlled supply of the datasets for the duration considered in this study. These stations are located in large homogenous fields to provide a good consistent record to compare with the satellite datasets and to reduce errors in the spatial mismatch between in situ and satellite datasets. Since the study area consists of 10 ground sites and each site can be considered equivalent to the nearest passive pixel, there are in total 160 passive pixels observed in 16 days. Out of these, observations of 7 stations in 16 days (112 pixels) were used to model calibration and the rest to assess accuracy.

## 3. Methods

### 3.1. Baseline Algorithm

*SLPRM* is developed to retrieve *VSM*, *LST* and *T_C_* from measurements at six frequency channels of *AMSR2* at H and V polarizations at frequencies 6.9 GHz, 10.65 GHz, 18.7 GHz in a simultaneous manner [16]. The steps of this algorithm are as follows:

Step 1. Computation of dielectric constant by applying the model presented by [18]. The ground measured *Bd* and initial values for *VSM* and *LST* are required, obtained based on their average amounts in the region, through Equation (1):

(1) ε=f(VSM,LST,Bd)

where *ε* is the real part of the complex soil dielectric constant.

Step 2. Estimation of effective land surface reflection, is obtained through Equation (2) introduced by Fresnel expressions. To model roughness (i.e., sig and cl parameters), the model presented by [19] is applied through Equations (3) and (4).

(2) $$\begin{array}{l} r_{H}={\left|\frac{\cos(\theta -\sqrt{\varepsilon -\sin^{2}(\theta )})}{\cos(\theta )+\sqrt{\varepsilon -\sin^{2}(\theta )}}\right|}^{2}\\ r_{V}={\left|\frac{\varepsilon .\cos(\theta -\sqrt{\varepsilon -\sin^{2}(\theta )})}{\varepsilon .\cos(\theta )+\sqrt{\varepsilon -\sin^{2}(\theta )}}\right|}^{2} \end{array}$$

(3) $$\begin{array}{l} \log\left[Q_{P}(f)\right]=a_{P}(f)+(b_{P}(f)\times \log(sig/cl))\\ +(c_{P}(f)\times (sig/cl)) \end{array}$$

(4) $$\begin{array}{l} R_{P}^{e}=(Q_{P})\times r_{P}+Q_{P}\times r_{q} \end{array}$$

where $R_{P}^{e}$ represents reflectivity, Fresnel reflectivity denoted by the term *r*, *θ* is incidence angle, *a*, *b* and *c* are the frequency-based coefficients. Parameters H and V are horizontal and vertical polarization, respectively. *p* represents the desired polarization and *q* represents the opposite polarization. *f* represents the frequency, *Q_P_* is related to surface roughness, which can be calculated for different frequencies in horizontal and vertical polarization.

Step 3. Effective reflectivity ($R_{P}(\theta )$) can be converted into effective emissivity ($E_{P}(\theta )$), through Equation (5):

(5) $$E_{P}(\theta )=1-R_{P}(\theta )$$

Step 4. Here the effective temperature is calculated through Equations (6) and (7) as shown below:

(6) $$T=\left(\left(1-\Gamma \right)\times T_{C}\right)+\left(\Gamma \times T_{S}\right)+\left(\left(1-\Gamma \right)\times \Gamma \times \left(1-E_{P}\left(\theta \right)\right)\times T_{C}\right)\;$$

(7) Γ=f((VI)×θ) 

where Γ is vegetation transmissivity, *VI* is vegetation index and *T*, *T_S_* and *T_C_* are effective temperature, temperatures of the soil and canopy temperature, respectively.

Step 5. Calculation of *BT* by applying the effective temperature and emissivity, Equation (8):

*BT_P_* = *E_P_* (*θ*) × *T* (8)

Step 6. Here, the obtained *BT* and sensor measured *BT* are compared through error minimization method with variables χ = {*VSM*, *LST*, *T_C_*} that minimizes χ^2^, through Equation (9):

(9) $$\chi^{2}=\overset{6}{\underset{i=1}{\sum }}[{\left({\left(BT_{obs}\right)}_{i}-{\left(BT_{est}\right)}_{i}\right)}^{2}]$$

### 3.2. Algorithm Improvement

#### 3.2.1. VWC Index Extraction

Since the *Qp* method is used in the *SLPRM* algorithm to model the roughness effects, the main purpose of this study is to minimize the effect of vegetation on the *AMSR2* microwave *BT*, by applying appropriate modelling for the retrieval of *VSM* content along with *LST* and *T_C_* from microwave data. Because the purpose is to aggregate the observations of passive microwave and optical sensors to estimate *VWC*, the two Multi Polarized Difference Index (*MPDI*) and Normalized Difference Water Index (*NDWI*) are used as the proper indicators of *VWC*, obtained from the *AMSR2* and *MODIS* sensors, respectively. The multi-polarization measurements at higher microwave frequencies are suitable for modelling the vegetation effects [11]. The *MPDI* derived from 36.5 GHz, as a good representative of the *VWC*, can be calculated through Equation (10):

(10) $$MPDI=\left(BT_{V}-BT_{H}\right)/\left(BT_{V}+BT_{H}\right)$$

where, *BT_V_* and *BT_H_* are the vertical and horizontal *BT* in passive microwave sensors, respectively.

Because *NDWI* is more consistent with *VWC* than other (*Vis*/*IR*) vegetation indices, it is calculated through Equation (11). Band 2 (850 nm, *NIR* band), and one of 5 or 6 bands (1240 nm or 1650 nm, *SWIR* band) of *MODIS* can be applied. In this study, band 5 is used as the *SWIR* band, because photons at 850 nm and 1240 nm penetrate into vegetation canopies in a similar manner, with similar atmospheric scattering [20].

(11) NDWI=(NIR−SWIR)/(NIR+SWIR) 

where, *NIR* is near infrared and *SWIR* is shortwave infrared.

Due to the different spatial resolution of *MODIS* and *AMSR2* sensors, *NDWI* is extracted from *MODIS* pixels are averaged to each passive pixel.

At this stage, the main issue is the integration of these two indices in development of new index called *POVI*, in a sense that it is able to model the vegetation effect at its best. Due to different high sensitivity of transmissivity to the *MPDI* and *NDWI*, the weights (wi) are considered to calculate *POVI* in the Equation (12):

(12) $$POVI=\overset{n}{\underset{i=1}{\sum }}w_{i}\times {\left(VI\right)}_{i}\;$$

Since the objective here is to calculate the vegetation transmissivity (*Γ*) through Equation (7) of step 4, it is necessary to examine the correlation between the vegetation transmissivity and the *VWC* indices. In this study, linear (first order) and nonlinear (exponential), both are applied to the vegetation transmissivity and the *VWC* indices as detailed in the following sections.

#### 3.2.2. Transmissivity Modelling

##### Linear, First Order

**Figure 2:**
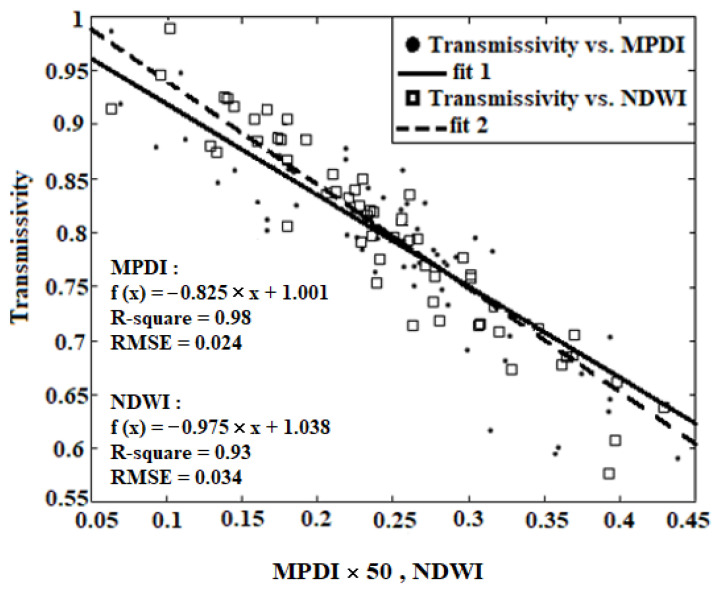
Scatter plots of *MPDI* and *NDWI* versus transmissivity using linear regression.

As observed in Figure 2, the *MPDI* is more consistent with transmissivity than NDWI; therefore, due to its lower Root-Mean-Square Error (*RMSE*) and higher contribution in estimating vegetation effect, it should weigh more.

In linear regression, the *R*^2^ is the best statistical parameter to determine the degree of compatibility and can be obtained through the weights calculated through Equation (13).

(13) $$w_{i}=\frac{{\left(R^{2}\right)}_{i}}{\sum^{\;}{\left(R^{2}\right)}_{i}}\;$$

**Figure 3:**
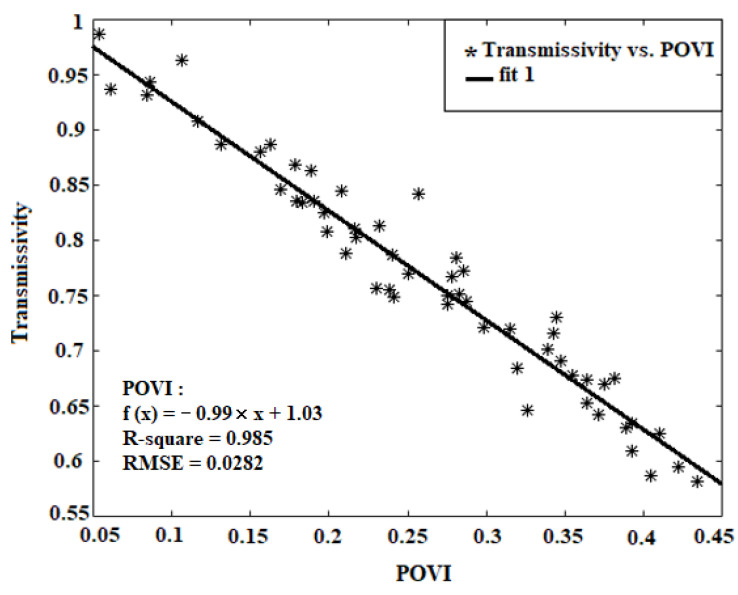
Scatter plots of *POVI*—versus transmissivity with linear regression.

Equation (14) is defined as a linear regression, applied to estimating vegetation transmissivity. The relationship between calculated *POVI* and vegetation transmissivity using linear regression is shown in Figure 3. The comparison between Figure 2 and Figure 3 indicates that the *POVI* is less compatible with the transmissivity than that of the *MPDI*:

(14) Γ=(a×(POVI))+b 

where *a* and *b* are the constant coefficients of this equation.

Calculations were made to find the weight and the coefficients of linear regression according to Table 2, to determine the vegetation transmissivity, the *SLPRM* algorithm is applied and compared against ground measurements.

##### Nonlinear, Exponential 

**Figure 4:**
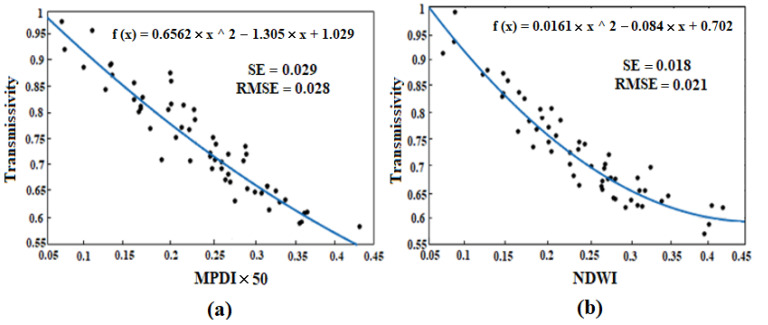
Scatter plots of *MPDI* (**a**) and *NDWI* (**b**) versus transmissivity.

The exponential correlations between transmissivity and both the *NDWI* and *MPDI* are shown in Figure 4. Hence, the nonlinear regression applied in estimating the vegetation transmissivity is expressed through Equation (15):

(15) Γ=exp(−a×VI−/cosθ) 

where *a* is a constant coefficient with a value of 100.

**Figure 5:**
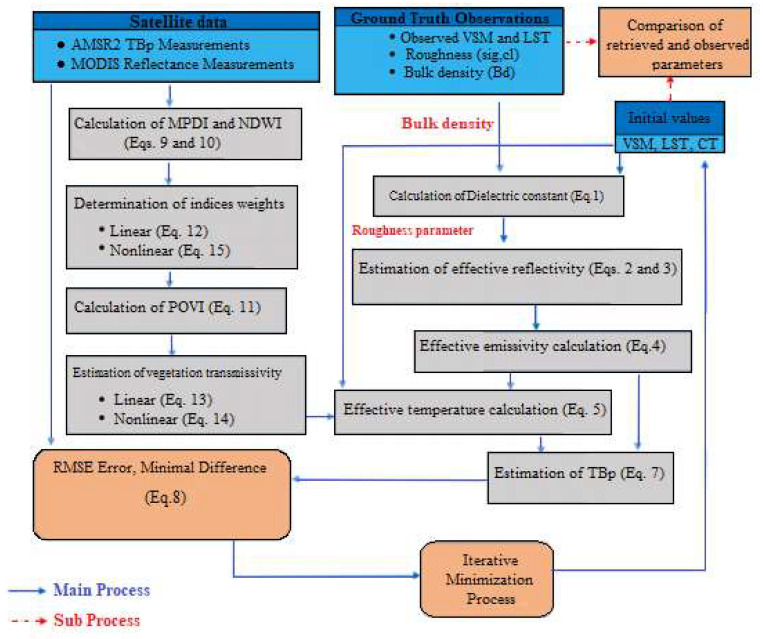
Workflow employed in this study.

As observed, there exists a correlation between Figure 4 and Equation (14). The indices weights are calculated through Equation (16):

(16) $$w_{i}=(1-\frac{SE_{i}}{\sum SE_{i}})\;$$

where, *SE* is the standard error. In Figure 5, a flowchart is provided for easier understanding of the algorithm and its modifications.

## 4. Results and Discussion

**Figure 6:**
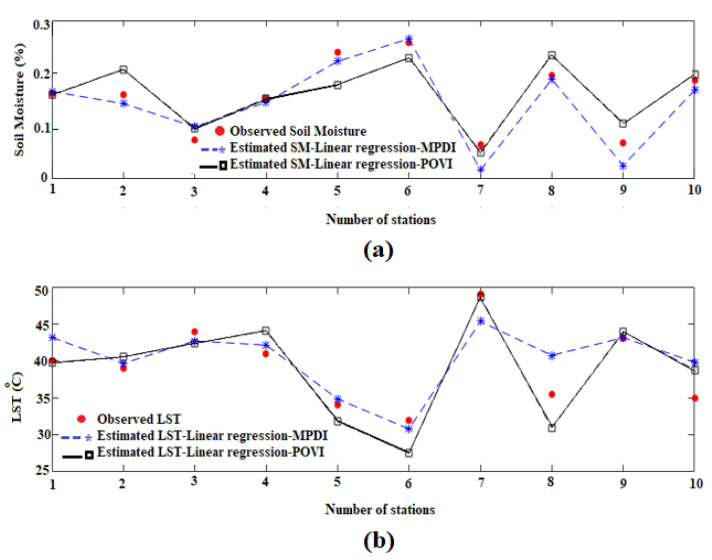
Comparison between observed and estimated Soil moisture (**a**) and Land surface temperature (**b**) parameters using *POVI* and *MPDI* in linear relationship.

Vegetation water content is estimated together with *VSM*, *LST* and *T_C_* through an improved physical model. In order to improve the surface parameters retrieval accuracy, the surface roughness is inserted in the *SLPRM* algorithm. In this study to improve the performance of this retrieval algorithm, the *POVI* is introduced and evaluated in estimating *VWC* and therefore, vegetation effects modelling. Here, the equations developed can estimate vegetation transmissivity from the *POVI*. Surface roughness (sig/cl) of constant amount is considered as 0.12, which is the average of regional roughness. As a general result, the comparison between the accuracies of *VSM* and *LST* retrieval through the two different linear and nonlinear regression can be made according to Table 2. The *RMSE* of *VSM* and *LST* retrieval based on the linear regression are also tabulated in Table 2. The findings related to the implementation of the linear regression with *POVI* and *MPDI* are provided in Figure 6. In this figure, a comparison is made between the observed and retrieved parameters in ten stations. As mentioned, the observations of the last three stations, which are the checkpoints, are used to estimate the retrieval accuracy. 

According to Figure 6 and as observed in Table 2, the *RMSE* of *VSM* and *LST* retrievals through linear regression and *POVI* are obtained as 0.044 (m^3^/m^3^) and 3.03 °C, respectively. While, if only the *MPDI* is applied, these values fall to 0.042 (m^3^/m^3^) and 2.91 °C, which indicates the better performance of *MPDI*. In the first where a linear correlation is applied between vegetation indices and transmissivity, there is a reduction in accuracy of the *VSM* and *LST*, compared with the condition where only *MPDI* is applied. Unlike the linear regression model, in the second case, the integration of indices increases the accuracy of the *VSM* and *LST*, as compared to *MPDI* and *NDWI*. 

**Figure 7:**
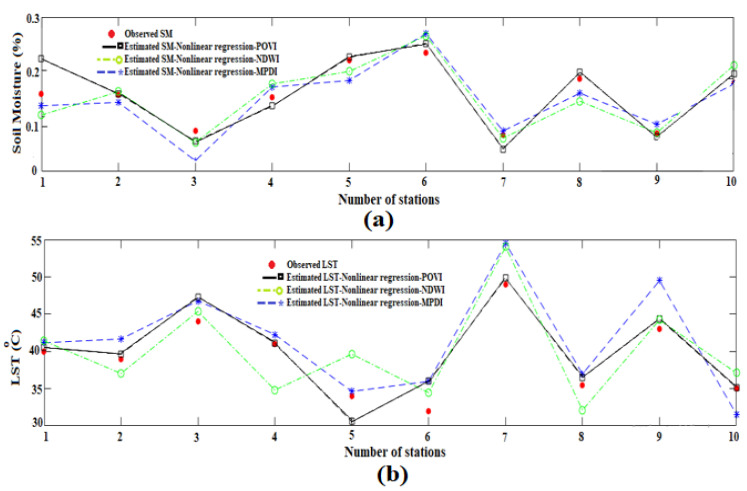
Comparison between observed and estimated Soil moisture (**a**) and Land surface temperature (**b**) parameters using *POVI*, *MPDI* and *NDWI* in nonlinear relationship.

In the nonlinear regression model, the weights and coefficients of nonlinear regression are calculated and given in Table 2, the *SLPRM* algorithm is applied in *VSM*, *LST* and *T_C_* retrieval. By applying the ground measurements, the overall accuracy of the retrieved soil parameter is estimated. The *RMSE* of *VSM* and *LST* retrieval based on the nonlinear regression are given in Table 2. A comparison between ground measurement, and retrieved parameters, where the nonlinear regression is applied based on *MPDI*, *NDWI* and *POVI*, are shown in Figure 7. As observed, the *RMSE* of retrieved *VSM* and *LST*, where the nonlinear regression is applied, are obtained as 0.038 (m^3^/m^3^) and 2.81 °C, respectively, with *MPDI*. If the *POVI* is applied, these values also fall to 0.031 (m^3^/m^3^) and 2.28 °C, which indicates the better performance of *POVI*. It should be noted that using the *NDWI* alone instead of the *MPDI*, also improves the accuracy of *VSM* and *LST* retrieval by about 0.05% and 0.49 °C. As observed in Figure 4, there exist exponential correlations between vegetation transmissivity and both *NDWI* and *MPDI*. This exponential relationship of *NDWI* is even more visible. For this reason, the insertion of the *NDWI* into the linear regression will weaken the performance of the algorithm and ultimately reduce the retrieval accuracy.

**Figure 8:**
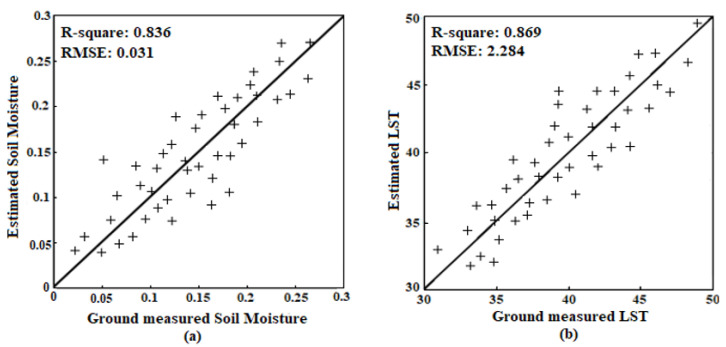
Comparison between measured and estimated parameters using *POVI* in nonlinear relationship: (**a**) Soil moisture (%), (**b**) Land surface temperature (°C).

While in nonlinear regression, the presence of the *NDWI* contributes to the better performance of the algorithm. In other words, the integration of *NDWI* with the *MPDI* in constructing the new vegetation index, i.e., *POVI*, increases the retrieval accuracy, only in nonlinear regression model. Moreover, a comparison has been made between measured and the most accurately estimated parameters, through nonlinear regression and *POVI*, at three check stations in 16 dates that shown in Figure 8. Note that, the in situ data are point measurements of soil moisture in the top 6 cm profile, whereas satellite provide measurement at some foot print, which may cause a spatial mismatch and some error in validation. However, in comparing with other methods [11,21,22], the *RMSE* values suggest that this improved *SLPRM* algorithm is sufficiently reliable to allow the estimates of all above mentioned parameters in the tested sites.

The accuracy of parameters retrieval is statistically improved when a nonlinear correlation is considered between *VWC* and vegetation transmissivity, as well as the aggregation of passive microwave and optical (*VIs*/*IR*) indices due to their sensitivity to the vegetation. It is more appropriate to apply passive microwave and optical (*VIs*/*IR*) observations on different platforms, like the *AMSR2* and the *MODIS* observations, as applied in this study [6]. In addition to proper modelling of the *VWC* effects on *BT*, considering the roughness in the *SLPRM* is one of the reasons for the algorithm’s success. The study reveals that the changes in surface roughness also influence the emissivity of natural surfaces, and therefore, its modelling in physical soil moisture retrieval algorithms is of great importance. Furthermore, due to the fact that different soil parameters are affected by each other, establishing a proper relationship between different parameters is the key to have more accurate retrievals. In this study, an algorithm for retrieving three parameters simultaneously is presented. Due to the possibility of observing two parameters of soil moisture and temperature, the accuracy of these two retrieved parameters has been evaluated. Although the ground observation of the canopy temperature parameter is not possible, simultaneous retrieval of this parameter along with two other parameters improves their retrieval accuracy.

## 5. Conclusions

In this study, soil moisture, canopy temperature and land surface temperature were obtained by applying the *SLPRM* model at different levels of vegetation density, validated by using ground sensor data like soil moisture and soil temperature. Through this study, an attempt has been made to integrate passive microwave *AMSR2* data and optical (*Vis*/*IR*) *MODIS*. Both linear and nonlinear models were tested for estimating the *VWC* indices and vegetation transmissivity. A new index called *POVI*, resulting from integration of two sensors data, has been developed for an improved *VWC* estimation and ultimately soil moisture retrieval.

The study showed that it is possible to estimate soil parameters with an improvement accuracy of about 20%, by applying different sensors for vegetation modelling in the *SLPRM* algorithm. Due to the possibility of extracting valuable information, this capability may be useful in climatic, agricultural, and soil moisture retrieval studies. This newly devised method allows the modelling of surface roughness and vegetation effects at *AMSR2* spatial scale in addition to retrieving reliable, acceptable and accurate soil parameters from satellite observations. Further studies in expanding this algorithm could focus on evaluation of soil parameters retrieval in specific regions (high vegetative areas) and testing other vegetation indices by inclusion of more sensors.

## Figures and Tables

**Table 1:** Details about the satellite datasets used in this study.

Source	Used Channels	Spatial Resolution	Temporal Resolution	Purpose
*AMSR2/GCOM-W*	6.9, 10.65 and 18.7 GHz (v and h polarizations)	25 km	Daily	*VSM*, *LST* and *T_C_* retrieval
*AMSR2/GCOM-W*	36.5 GHz (v and h polarizations)	25 km	Daily	Derivation of *MPDI* (calculation of vegetation water content)
*MODIS/Aqua*	Band 2 (850 nm, NIR), Band 5 (1240 nm, *SWIR*)	1 km	Daily	Calculation of *NDWI*

**Table 2:** VSM- and -LST- retrieval accuracies.

Regression Type	Parameters	Coefficients	Type of INDEX	RMSE VSM (m^3^/m^3^)	RMSE LST (°C)
Linear regression	*a* = −0.041	*w*_1_ = 1, *w*_2_ = 0	*MPDI*	0.042	2.92
	*b* = 0.984	*w*_1_ = 0.513, *w*_2_ = 0.486	*POVI*	0.044	3.03
Nonlinear regression	*a* = 100	*w*_1_ = 0.38, *w*_2_ = 0.62	*POVI*	0.031	2.28
		*w*_1_ = 0, *w*_2_ = 1	*NDWI*	0.033	2.32
		*w*_1_ = 1, *w*_2_ = 0	*MPDI*	0.038	2.81

## Data Availability

All the datasets used in this study are freely available.

## References

[1] Sun F., Lü Y., Wang J., Hu J., Fu B. (2015). Soil moisture dynamics of typical ecosystems in response to precipitation: A monitoring-based analysis of hydrological service in the Qilian Mountains. Catena.

[2] Entekhabi D., Reichle R.H., Koster R.D., Crow W.T. (2010). Performance metrics for soil moisture retrievals and application requirements. J. Hydrometeorol..

[3] Walker J.P., Houser P.R. (2004). Requirements of a global near-surface soil moisture satellite mission: Accuracy, repeat time, and spatial resolution. Adv. Water Resour..

[4] Srivastava P.K. (2017). Satellite soil moisture: Review of theory and applications in water resources. Water Resour. Manag..

[5] Chanzy A. (1993). Basic soil surface characteristics derived from active microwave remote sensing. Remote Sens. Rev..

[6] Li Q., Zhong R., Huang J., Gong H. (2011). Comparison of two retrieval methods with combined passive and active microwave remote sensing observations for soil moisture. Math. Comput. Model..

[7] Njoku E.G., Chan S.K. (2006). Vegetation and surface roughness effects on AMSR-E land observations. Remote Sens. Environ..

[8] Colliander A., Chan S., Kim S.-b., Das N., Yueh S., Cosh M., Bindlish R., Jackson T., Njoku E. (2012). Long term analysis of PALS soil moisture campaign measurements for global soil moisture algorithm development. Remote Sens. Environ..

[9] Srivastava P.K., Han D., Ramirez M.A., O’Neill P., Islam T., Gupta M. (2014). Assessment of SMOS soil moisture retrieval parameters using tau-omega algorithms for soil moisture deficit estimation. J. Hydrol..

[10] Gao Z., Xu X., Wang J., Yang H., Huang W., Feng H. (2013). A method of estimating soil moisture based on the linear decomposition of mixture pixels. Math. Comput. Model..

[11] Owe M., de Jeu R., Walker J. (2001). A methodology for surface soil moisture and vegetation optical depth retrieval using the microwave polarization difference index. IEEE Trans. Geosci. Remote Sens..

[12] Santi E., Pettinato S., Paloscia S., Pampaloni P., Macelloni G., Brogioni M. (2012). An algorithm for generating soil moisture and snow depth maps from microwave spaceborne radiometers: HydroAlgo. Hydrol. Earth Syst. Sci..

[13] El Hajj M., Baghdadi N., Bazzi H., Zribi M. (2019). Penetration analysis of SAR signals in the C and L bands for wheat, maize, and grasslands. Remote Sens..

[14] Petropoulos G., Srivastava P.K., Ferentinos K.P., Hristopoulos D. (2020). Evaluating the capabilities of optical/TIR imaging sensing systems for quantifying soil water content. Geocarto Int..

[15] Yang S., Shen S., Li B., Le Toan T., He W. (2008). Rice mapping and monitoring using ENVISAT ASAR data. IEEE Geosci. Remote Sens. Lett..

[16] Moradizadeh M., Saradjian M.R. (2016). The effect of roughness in simultaneously retrieval of land surface parameters. Phys. Chem. Earth Parts A/B/C.

[17] Mojiri A., Jalalian A. (2011). Relationship between growth of Nitraria schoberi and some soil properties. J. Anim. Plant Sci..

[18] Dobson M.C., Ulaby F.T., Hallikainen M.T., El-Rayes M.A. (1985). Microwave dielectric behavior of wet soil-Part II: Dielectric mixing models. IEEE Trans. Geosci. Remote Sens..

[19] Shi J., Jiang L., Zhang L., Chen K.-S., Wigneron J.-P., Chanzy A. (2005). A parameterized multifrequency-polarization surface emission model. IEEE Trans. Geosci. Remote Sens..

[20] Gao B.-C. (1996). NDWI—A normalized difference water index for remote sensing of vegetation liquid water from space. Remote Sens. Environ..

[21] Saradjian M., Hosseini M. (2011). Soil moisture estimation by using multipolarization SAR image. Adv. Space Res..

[22] Zhao T., Shi J., Bindlish R., Jackson T., Cosh M., Jiang L., Zhang Z., Lan H. (2015). Parametric exponentially correlated surface emission model for L-band passive microwave soil moisture retrieval. Phys. Chem. Earth Parts A/B/C.

